# Feasibility and Acceptability of a Mobile Technology Intervention to Support Postabortion Care After Surgical Abortion (the FACTS Study Phase 3): Mixed Methods Prospective Pilot Study

**DOI:** 10.2196/46284

**Published:** 2024-01-09

**Authors:** Roopan Gill, Gina Ogilvie, Wendy V Norman, Brian Fitzsimmons, Ciana Maher, Regina Renner

**Affiliations:** 1 Department of Obstetrics and Gynecology University of Toronto Toronto, ON Canada; 2 Women's Health Research Institute University of British Columbia Vancouver, BC Canada; 3 Department of Obstetrics and Gynecology University of British Columbia Vancouver, BC Canada; 4 Department of Family Practice University of British Columbia Vancouver, BC Canada

**Keywords:** mobile health, mHealth, digital health, abortion, human-centered design, sexual and reproductive health, qualitative research, mixed methods

## Abstract

**Background:**

In Canada, 1 in 3 women and people of gestational age undergo an abortion in their lifetime. Despite the liberal legal context, barriers continue to exist for women and people who can become pregnant to access this service.

**Objective:**

This study aims to (1) conduct a pilot study to demonstrate the feasibility and acceptability of myPostCare to support follow-up care after a procedural abortion; (2) use the findings to understand whether myPostCare has the potential to improve contraceptive behavior and knowledge, emotional well-being, and sexual health knowledge; and (3) develop a better understanding of how innovative mobile solutions can support integrative health programs in British Columbia with the goal of expanding to other sites across Canada.

**Methods:**

People of gestational age (aged 14-45 y) who underwent a procedural abortion were recruited from 2 urban abortion facilities in British Columbia. The participants completed a baseline quantitative survey and were provided access to myPostCare for up to 30 days. A follow-up quantitative survey was sent via email on day 30. Qualitative interviews were conducted to explore user satisfaction and usability of myPostCare. Responses to the survey questions were summarized using descriptive statistics, and the system usability scale (SUS) was scored according to the instructions. A secure analytics platform was implemented to obtain data on the overall use of the website by users. Qualitative analysis was conducted with NVivo using a thematic analysis approach. This study was approved by the Women’s and Children’s Research Ethics Board.

**Results:**

Overall, 62 participants were recruited (average age 30 y); 40% (25/62) of the participants completed the exit surveys, and 24% (6/25) consented to participate in the semistructured interviews; 40 participants had undergone an immediate postabortion intrauterine device (IUD) insertion, and 22 did not have an IUD inserted. Participants were satisfied with myPostCare. The SUS average score was 81.5 (SD 9.7; median 82.5, IQR 77.5-87.5), indicating high usability of the tool. Overall, 88% (22/25) of the participants changed their contraceptive method to an IUD. Web-based analytics demonstrated that there were 61 unique visitors to the site, and the top pages visited were Postprocedure Care, Emotional Well-Being, and Contraception Explorer. The longest time spent on the website was 56 minutes. The overall email open rate was 80%, with a click rate of 36%.

**Conclusions:**

This study demonstrates that communities and individuals are important collaborators in developing a mobile innovation that facilitates access to high-quality patient-centered abortion care. Through the cocreation process, a digital platform such as myPostCare highlighted a gap in abortion care in Canada, particularly around follow-up support after a procedural abortion.

## Introduction

More than 30% of women and people who can become pregnant in Canada undergo an abortion. To support access to this essential service, Canadian federal legislation requires that abortion services be provided by each provincial and territorial health system [1,2]. Recent literature suggests that despite the prevalence of the procedure, stigma surrounding abortion in Canada leads to barriers for women and people who can become pregnant to access postabortion support and share their abortion experiences [3]. The provision of information is an essential part of good-quality abortion services, which include follow-up care after a procedural abortion [4,5]. According to the National Abortion Federation Clinical Policy Guidelines, “appropriate and accessible post-procedure and follow-up care is essential to patients’ wellbeing” [4]. There is little evidence to suggest that “mandatory” follow-up visits will detect conditions that women and people who can become pregnant cannot detect themselves; however, there is evidence to suggest that more novel methods of offering “follow-up” visits for postabortion support are desired [6]. This includes the use of innovative mobile health (mHealth) solutions defined by the World Health Organization as “the use of mobile and wireless technologies to support the achievement of health objectives” [6,7]. More importantly, given the potential reach of mHealth, evidence has pointed to its potential to provide remote support and follow-up, particularly for women and people who can become pregnant and live in rural and remote areas [8].

In Canada, geographic barriers impact abortion access, leading women and people who can become pregnant to travel long distances for services. Upon returning to their communities, they may face challenges in accessing minimal, ineffective, or nonexistent follow-up care [8,9]. A qualitative study further explored women’s abortion experiences in the Yukon territory, a remote Canadian service area, highlighting that “fragmented services left women unsatisfied, stressed, and upset about lack of information, multiple appointments, and lengthy wait times” [10]. Women further expressed frustration with lack of follow-up counseling and recommended that it be routinely offered as they feel contact with health care providers is cut off after the procedure [10]. In addition to access issues, barriers of cost, knowledge among the general public, and health care provider competence and attitudes have also been highlighted in the literature [9]. Another study explored women’s expressed desire for postabortion support services, highlighting the stigma surrounding abortion that exists in political and social contexts, preventing women from sharing their experiences [3]. This study specifically highlighted that although women may not necessarily need mandatory physical follow-up, they desire access to postabortion support for emotional well-being [3]. Furthermore, there is a great deal of inconsistency in the type of support and information available to women and people who can become pregnant after an abortion.

The *New England Journal of Medicine* published a special report on Telehealth in the United States, highlighting its utility and future. In 2016, Kaiser Permanente of Northern California reported that its virtual (email, telephone, and video) communications had exceeded in-person visits [11]. Similarly, research supports the safe and effective use of telehealth for the provision of medication abortion care globally [12-14].

Three trials of mHealth interventions have aimed to study the role of mobile interventions in increasing the use of contraception [15-18]. Mobile for Reproductive Health and Mobile Alliance for Maternal Action have used best practices from health communication programs to systematically develop family planning text messages [18]. Furthermore, Smith et al [13,14] explored women’s needs in Cambodia to develop a mobile phone–based intervention to support postabortion family planning, specifically contraceptive adherence. In the United States, research on the acceptability and feasibility of remote technologies for follow-up after medication abortion suggested that women prefer either a telephone call or a text message over a clinic visit [19]. Most recently, researchers from University of San Francisco’s Program in Women-Centered Contraception developed a tablet-based contraceptive decision support tool for women [20]. This study used a multiphase approach that incorporated the end user throughout the entire design of the project. The tool has been designed in collaboration with key stakeholders and designers from Bedsider [21]. Using an iterative process informed by patient and provider input throughout, this family planning innovation demonstrated that including users in development led to a more patient-centered innovation [22]. Despite the development and implementation of these mHealth innovations for family planning, research is limited in understanding the follow-up needs of women and people who can become pregnant and undergo an abortion, and how they would perceive a tool to support them and to engage them as active participants in the design process.

Given the existing evidence in support of mHealth for family planning innovations, we aimed to determine whether a mobile technology intervention would be acceptable and feasible for women and people who can become pregnant to support follow-up care after first or second trimester procedural abortion. We developed a 3-phased study based on human-centered design and the “Development-evaluation-implementation” process from the Medical Research Council Frameworks for Complex Interventions [23] rooted in 2 theories: Technology Acceptance Model and Theory of Reasoned Action [24,25] phases 1 and 2 have been published previously [26,27]. This study was a prospective pilot that aimed to determine whether the intervention was satisfactory, acceptable, and usable for women and people who can become pregnant to support them in follow-up after a procedural abortion. Ultimately, this study is the first to use mHealth and human-centered design in Canada as a novel approach to support follow-up care for women and people who can become pregnant and undergo procedural abortion.

## Methods

### Participants

Participants were recruited from 2 publicly funded abortion clinics in British Columbia, Canada. The eligibility criteria were as follows: (1) consent to undergo a first or second trimester procedural abortion, (2) ability to read and write English, (3) ability to participate in study procedures, and (4) aged ≥14 years. Participants were excluded if they were (1) attending the clinics because of fetal anomaly or miscarriage, (2) undergoing medication abortion, (3) in a situation where it may be dangerous to use a mobile intervention, and (4) unable to provide consent to participate. To elicit whether a woman was in a dangerous situation, counselors asked the patients as part of routine care if they felt safe in their current relationships. In cases where a risk is identified, counselors provided resources and would refer to the appropriate provider or service.

### Study Design

The overall study design is a mixed methods user-centered design approach with 3 phases based on the “Development-evaluation-implementation” process from the Medical Research Council Frameworks for Complex Medical Interventions [28]. This is the final phase of the 3-phase study, with the findings from phases 1 and 2 already published [26,27]. Phase 3 is a prospective pilot mixed methods study conducted in 2 urban clinics in Vancouver, British Columbia, between March and June 2018 to test the acceptability and feasibility of myPostCare when implemented as part of clinical care. This study was approved by the Children’s and Women’s Research Ethics Board (H18-00036).

Eligible participants were screened by a primary investigator. They were then introduced to the study and consented under the supervision of the research coordinator. Participants consented to be contacted for a qualitative interview at 4 weeks. A baseline questionnaire that was adapted from validated survey tools was filled out to collect demographic information, contraception history, and levels of perceived well-being and distress in the past 2 months before the abortion [29-31]. The Arizona Integrative Outcomes Score was used and is a validated 1-item visual analog tool that allows self-rated global assessment of spiritual, social, mental, emotional, and physical well-being over the past 24 hours and 1 month [32].

The participants were registered on the website at the end of each recruitment day. Participants received 7 automatic email notifications that were timed with what would be expected after the procedure and prompted them to the website over the course of 30 days. At the end of 30 days, participants received a link to their email to complete a questionnaire adapted from the validated questionnaires [29-31,33,34]. This questionnaire specifically included questions about satisfaction with myPostCare, a system usability scale (SUS) comprising 10 questions, and an evaluation of the impact of various aspects of myPostCare including emotional well-being, contraceptive behavior, immediate postprocedural care, and sexual health. Data analytics were collected using a secure data analytic platform housed at the BC Children’s Hospital. Participants were compensated for their participation.

Participants who consented to the qualitative part of the study were contacted and invited to participate in semistructured interviews to explore their engagement with the mobile tool. This included system usability, experience of receiving email notifications, emotional well-being, contraceptive decision-making, immediate postprocedural concerns, and questions about sexual health. Questions explored experience with receiving timed email messages, feedback on the content of the notifications themselves, if they found the notifications helpful and why, did they follow the recommendations of the notifications, and did they visit the website after being prompted by the notifications. Participants received additional compensation for their participation in the interview.

### Data Analysis

Descriptive analysis of each variable from the quantitative surveys and secure data analytic platforms was reported as mean (SD) or median for continuous variables and count (percentage) for categorical variables. All statistical analyses were performed in R (R Foundation for Statistical Computing). Using Piwik, a secure web analytics through the BC Children’s Hospital Research Institute, specific user engagement data were gathered from February 20 to May 2, 2018. The semistructured interview transcripts were uploaded to NVivo 11 (Lumivero) and read by 2 researchers. Inductive analysis was performed to identify emerging themes that were further refined through collaborative analysis with the first author and coinvestigator [35]. The highlighted text was coded into nodes representing similar or repeated ideas. Some text was coded to >1 node, reflecting the number of ideas presented. The nodes were categorized into specific themes, forming a thematic map that was later discussed with the research team. To enhance the validity of the findings, a triangulation approach was used. This involved cross-referencing data from the quantitative survey and the subsequent 2 phases of this study.

### Ethical Considerations

This study received ethics approval by the Children’s and Women’s Research Ethics Board (H18-00036). Informed consent was obtained from all the participants included in this study. The study data were anonymized and deidentified. All data were stored in an encrypted file only accessible to the research team involved in the analysis of the study. Compensation was not provided to those who had completed the survey. A CAD $25 (US $18.38) honorarium was provided to those who completed an interview.

## Results

### Participant Characteristics

Participants were recruited from 2 abortion clinics in Vancouver, British Columbia. A total of 62 participants were recruited and completed the baseline survey. Of the 62 participants recruited, 25 (40%) women and people who can become pregnant responded to the follow-up survey. We investigated whether systematic differences existed between women who responded and those who did not. Table S1 in Multimedia Appendix 1 provides a summary of the demographic information from the baseline survey and a comparison between responders and nonresponders. There were no substantial differences between these 2 groups for any of the variables listed, although there was a nonsignificant trend for the responders to have a lower Arizona Integrative Outcomes Score. These results were not statistically significant (*P*<.05). All the participants identified as ciswomen.

For the qualitative interviews, of the 25 participants who completed the exit survey, 6 (24%) consented to participate in semistructured individual interviews. These were conducted via telephone.

### Quantitative

#### Change in Contraceptive Method

Most of the respondents (22/25, 88%) indicated that they had changed their contraceptive method to an intrauterine device (IUD) at the time of their abortion, and 21 (95%) of the 25 respondents indicated that they had changed to a Mirena, whereas 1 (4%) of the 25 respondents indicated changing from a copper to Mirena. The contraceptive method of choice was not influenced by the website; however, the website and email notifications helped reassure participants about the signs, symptoms, and effectiveness of the IUD.

#### System Usability Scale

The SUS comprised 10 questions [36]. The average SUS was 81.5 (SD 9.7), and the median was 82.5 (IQR 77.5-87.5), which revealed that 75% (19/25) of the respondents indicated an SUS score >77, which is a very high score.

#### Satisfaction

**Figure 1:**
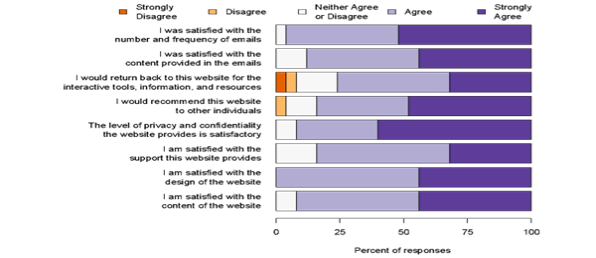
Graphical representation of the satisfaction results in percentage.

Most of the respondents were satisfied with the website. Figure 1 graphically displays these results as percentages.

### Qualitative

#### Overview

Qualitative analysis of the interviews was completed using thematic analysis including both inductive and deductive themes. Nine key themes were identified and are listed in Textbox 1.

**Key themes.**

**Theme**

1. Ease of use
2. Usefulness of myPostCare
3. Website
4. Frequency of use
5. Time spent on the website
6. Suggestions for improvement
7. Recommend to friend
8. Privacy and security
9. Design features
10. Overall impressions of myPostCare

#### Ease of Use

Overall, there was unanimous agreement that myPostCare was easy to use with an organized and easy-to-navigate design. One participant highlighted the following:

I thought it was very easy to use, which I really liked. I felt the information was well laid out with the menu sidebar on the side. The writing was easy to interpret and was not overly scientific. It was easy to navigate throughout the whole website. It didn’t feel like I was reading a research article. It was nicely spaced out and got to the point very quickly.

participant 3

Furthermore, the language was accessible and user friendly. The participants felt that the drop-down features were very effective. We added this feature after the usability testing from phase 2, and therefore, it was consistent among our participants to hear that they appreciated this feature. The participants were highly satisfied with the ease of use of myPostCare. When asked about ease of use as it pertained to the information, 1 participant shared the following:

The information there was superb. It was very user-friendly. Anyone could use that and get what they were looking for, no problem.

participant 5

#### Usefulness of myPostCare

We asked about the overall usefulness of myPostCare by asking separately about the website and email notifications. Participants were satisfied with the overall frequency and timing of the email notifications:

I like there was one email per week, it was not overwhelming. It gave you time to go back to the website in increments, not getting overwhelmed and not having it constantly on your mind, but it was a good refresher every week. This is what I needed.

participant 3

The timing was impeccable when you would get these e-mails and what you would be feeling. When they would come, they were right on point. I always felt like someone was at my fingertips if I needed help.

participant 5

They found that the emails helped to navigate the recovery process from immediate signs and symptoms, emotional well-being, and contraception decision-making to general sexual health, such as a better understanding of their menstrual cycle. They also found that the emails helped them feel supported and not alone. This was an important point that resonated with all participants interviewed. A few participants stated the following:

When I would get the email it would say, “Okay, now you might be going through this and this and this,” it gave me a moment to be like, “Right, I am. I might be going through this. I’m still having some symptoms. How am I actually feeling?” It was a reminder to check in with myself and also to think about how I may be experiencing symptoms at that time.

participant 1

It was nice to feel as though there was “someone” checking up on you even though it wasn’t a person. There was new content with each e-mail and helped to direct you to different stages of recovery process. I found that helpful.

participant 1

The participants unanimously stated that myPostCare provided them the support that was needed at the right time. It was helpful for the resource to provide support over time and that it allowed them to navigate various aspects of their postcare journey. A few participants shared the following:

I felt like I was cared for. It was amazing to get, “Hey, I hope you’re doing okay. Take care.” It just felt that someone was there for me and saying if you need to call or anything, you can at any time.

participant 2

I think this is a great resource. It was a really beneficial thing for me to have, for sure.

participant 6

#### Website

Overall, all participants stated that they did not have a favorite page but that each category was helpful depending on the stage at which they were in the recovery process. Each participant mentioned that the Postprocedure care page and the Emotional Well-Being Support tool were the most effective:

The emotional well-being tool was helpful. I liked how each emotion had a little blurb about. I liked the meditation.

participant 2

Talking about various emotions that occur was important because I found that one week I felt one way but then all of a sudden I would feel different. It was nice to go back to the website, have those feelings identified and made me feel normal.

participant 3

Most found that the emails were well timed with the website, and providing links embedded within the emails to direct participants to the website was appreciated. Participants stated that they did not click on the emotion “Good” but did use the suggestions provided such as the meditation, journaling, and going for a walk:

I wrote an entire journal entry one day, and that was really good and definitely got some crying out while I was doing that, so I think the website prompted me to do that that day, yes.

participant 3

Just going through and trying to be at one with this, checking in, using the tools. There was good days, bad days. I have a wonderful program at work as well but I didn’t have to reach out to it because there was stuff here about meditation and making sure that I am looking after myself and doing something nice for myself.

participant 5

In addition, a participant commented that the website had credible information, which helped to answer questions that she would have seen her physician about and, therefore, kept her out of the office or emergency room. When further asked if the website helped her understand when to seek hospital care, she mentioned that it was very clear. She specifically found the disclaimer useful in preventing women from misunderstanding the website as a substitute for clinical care:

It [myPostCare] kept my husband and I out of emergency rooms...Here we are, two weeks and three days, and all of a sudden there’s an email about IUDs being that you could have spotting for three to six months. I am like, “Okay. We are good.” Then the bleeding stopped. It was just very empowering to have that information.

participant 5

Finally, some participants found the website useful to support them as they did not have anyone else to talk to about their abortion, and the website helped them not feel isolated:

I went every time I got an e-mail and then there were two times that I went on it by myself when I was feeling pretty emotional and the emotional well-being tool helped.

participant 2

It was good for me because I didn’t tell anybody. I didn’t have anyone to talk to.

participant 4

It [myPostCare] is so critical, and I hope it never goes away and that it’s there for as long as women need this procedure. I hope that this site is always there. It was truly instrumental to my whole well-being through this whole procedure, so I thank you.

participant 5

#### Frequency of Use

Participants used the website on its own but also clicked on the links within the emails. Some participants saved all the emails so that they could return to them. Using the website for 1 month seemed to be sufficient for all participants. One participant stated that she had visited the website 10 to 15 times:

I went every time I got an e-mail and then there were two times that I went on it by myself when I was feeling pretty emotional and the emotional support tool helped.

participant 2

#### Time Spent on Website

The time spent on the website, as expressed in the qualitative interviews, was consistent with the results from the web-based analytics presented in the *Web-Based Secure Analytics* section. Most participants stated that they were on the website for anywhere from 2 minutes to 1 hour:

I’d probably went all together 10 to 15 times. There was one time I was on it for probably an hour, but the other times, it was probably anywhere between two to five minutes.

participant 2

I’d say around half an hour.

participant 3

Maybe the first couple of weeks, I kind of looked at it. I looked through it for half an hour at dinner, 20 minutes, 15, half an hour.

participant 4

...going to guess at least an hour going back through, making sure I didn’t miss anything going to the link, so at least an hour.

participant 5

#### Suggestions for Improvement of myPostCare

There was a strong sentiment to include blogs and stories shared by women and people who can become pregnant and have undergone an abortion. This was available on the website, but these were found under the “Good” emotion. Many did not necessarily explore this section and mentioned that if they were feeling good, they did not necessarily explore the emotional support tool and would be keener on using the Contraception Explorer or Sexual Health pages. Furthermore, suggestions to add these on the main landing page or to have rotating articles that are specific to women and people who can become pregnant telling their stories would be very useful. This was highlighted by participants as a means of further enhancing the community feeling and not feeling alone in their experience. When further explored, this also highlighted that sharing stories was also a way to help destigmatize the experience that many women and people who can become pregnant and who participated in our interviews had internalized. The following excerpts highlight this theme:

I really wanted to hear someone’s story that was positive. I would have liked to listen to just having a couple of people’s stories and how it affected them just to compare myself to them. I don’t know.

participant 2

Putting up videos or even having articles on different stories.

participant 2

For me personally, if I’m feeling good or when I was starting to feel good about myself again, I wouldn’t have gone on the website to check that.

participant 2

I think more testimonials and more quotes that you can use on that website from people who have been through the experience, the better because it gives validation for what women are going through and kind of makes us feel less alone.

participant 6

The more testimonials and the more feedback you can get from women of all ages, all experiences, all the better.

participant 6

#### Recommend to a Friend

All participants who were interviewed would recommend myPostCare to a friend. Some also suggested that this would be specifically good for friends who did not necessarily feel comfortable going to their physician or who did not have a family physician with whom they had a trusting relationship:

Yes, I think I totally would. I think it’d be very helpful to have. I don’t think it’s going to solve a friend’s problems or anything, but for me, it was helpful to have.

participant 2

Yes, and it’s definitely one that I want to, like, if I ever know somebody that is going through that, I’m definitely going to recommend that to them.

participant 3

I really would. This is a great resource for the person that doesn’t think that their issues always warrant a call to their doctor.

participant 6

#### Privacy and Security

Our participants were satisfied with the level of privacy and security afforded by the emails and website. In particular, they noted that the emails were separate from the website, and some participants suggested that it would be essential to keep it this way when myPostCare would be made live. One participant stated the following:

It’s very discrete, and I liked that. The login is required to get in on the website, so to me, it was certainty sufficient. My name is not all over the website, so even if I left it open, it is what it is, who knows what I was in. It’s not too specific so I was never worried if I had it open in public.

participant 5

#### Design Features

All participants stated that the design was professional and the language was unbiased. Many participants commented that the design of the website and emails was calming and supportive. They also enjoyed the consistency between the website and email notifications. Words such as “clearly thought out,” “pleasant and cool pictures,” “nice blues and greys,” and “positive and well-crafted” came up frequently among our interview participants. One participant commented the following:

Overall, I don’t know if this is weird to say, but it was very calming. Approachable in a sense. It doesn’t hurt your eyes to stay on the website for a while. I really liked the colours. The layout was easy.

participant 6

Other participants noted that the site was structurally thought through and that the design was relatable to them. One participant highlighted the following:

I thought it was clearly thought out and structurally too. The language is nicely worded and was very unbiased.

participant 2

It was very nice and pleasant, the pictures were very cool. I liked the ranges of things that were on there, the whole thing about meditating and then also just needing actual straight up information was really helpful too.

participant 2

Very soothing colours. The nice blues and then greys, yes.

participant 2

I thought it was very easy to use. I felt that the information was well laid out with the menu sidebar. The images were quite big and spaced out so had to scroll quite a bit and not get through a lot of information.

participant 3

There was no harsh colours. There was no in-your-face type of things that popped into the website. I liked there were no advertisements. I think the peaceful colourings, the “click this if you feel called to.” It’s nice to have that sense of well-being with positivity on a sensitive topic, it was well crafted.

participant 3

#### Overall Impressions of myPostCare

Overall, the participants were satisfied with myPostCare. They felt supported by the resource. There were very strong sentiments that this went above and beyond what they had expected. One participant stated that she was surprised to have such a good experience with a website, as she had never had such an experience. Some of the participants used the site with their partners and appreciated the section that was specifically for partners. One participant stated that the emails and website helped to keep her out of the emergency department, as it highlighted the normalcy of postprocedural recovery. In general, all participants felt that it was a great experience to have this resource, and many participants expressed that this resource should be available as long as women need abortions:

I really liked the resources simply because it went beyond just what we went through. Yes, I think that was one of my favorite or one of the things that when I got to, I was like, “Okay, there are crisis lines, and there are counselors.” Yes, of course, that’s what I expected to be on there. It went past that. It went to sexuality. It went to LGBTQ, or it went through different topics, so I feel like it was good education beyond what I just went through.

participant 2

Yes, I was really happy I signed up for it and I was getting those e-mails weekly. I was able to access it, once again, read about different perspectives. I think there were some things that I felt like it was only me or it wasn’t normal, and then it would say something on the website that would make me feel better, more calm.

participant 2

I would just grab my phone and then just go, look at the thing and, “Okay, this is normal to feel like this.” I don’t know if I had a favourite part, but I just found that everything was useful.

participant 4

It was my other rock. My husband was my one rock, and the other one was this. It knew when things were going to happen, and when I was panicking about things, all of the sudden, there will be an e-mail. It was just perfect timing, and it was amazing. It truly was. I felt like I wasn’t alone. I went through every link. Even the links that were outside the website, I checked out every one of them. I read stories. It brought a sense of calm to me, I guess. It was truly, I never had such a good experience off of a website like this one. It was amazing. My husband went through everything. You would be panicking. I don’t know how many times we went back to this website to make sure that something that was going on wasn’t out of the ordinary, and of course, there would be, that it wasn’t out of the ordinary, so it was amazing. It truly was.

participant 5

No, I think overall it was pretty straightforward. There wasn’t anything that I was surprised to see, and there wasn’t anything that I can remember that didn’t kind of fit in with what was expected through the e-mails. It all kind of made sense.

participant 6

I think it was just a great experience to trial the website. I have my own personal reasons for my procedure and how I came about doing so, but I think it’s a great source for people that want to have that sense of community. I think it works really well for the specific areas that you’re trying to find more clarity.

participant 6

### Web-Based Secure Analytics

Table 1 presents the analytics results. Specifically, of the 62 participants, the number of unique visitors on the website was 61 (98%). Although only 25 participants completed the exit survey, all participants except 1 (98%) visited the website at least once. The number of returning visitors was 42. The average daily page views were 5; the total number of page views through the study period was 432; the highest number of hits at a single visit was 35; and the top 3 pages were Postprocedure Care, Emotional Well-Being, and Contraception Explorer. In total, 75% (47/62) of the participants were mobile users and 25% (16/62) were desktop visitors. The most popular contraceptive page visited was the IUD. The details of the number of page views throughout myPostCare are presented in Table 2.

**Table 1:** myPostCare web-based analytics for user engagement from February 20, 2018, to May 2, 2018 (N=62).

		Web-based analytics data
Unique visitors to the website, n (%)		61 (98.4)
Average time spent on the website by visitors		1 min and 28 s
Longest visit on the website		35 hits
Total number of page views, n		432
Average daily page views, n		5
Participants who are mobile users, n (%)		47 (75)
Participants who are desktop visitors, n (%)		16 (25)
Participants who visited the Emotional Well-Being page, n (%)		28 (45)
Participants who visited the Contraception Explorer page, n (%)		27 (43)
Participants who visit the Postprocedure Care page, n (%)		46 (74)
Participants who visit the Sexual Health page, n (%)		21 (33)
Top 3 pages on the website		Postprocedure Care, Emotional Well-Being, and Contraception Explorer
**Most popular contraceptives visited from the contraception tool in page views, n**		
	Hormonal IUD^a^	13
	Sterilization	7
	Copper IUD	3
	Vaginal ring	3
	Fertility awareness	2
	Patch	2
	Abstinence	1
	Depo shot	1
	Female condom	1
	Male condom	1
	Withdrawal	1
**Visits to given feelings (good, okay, and not so good) from the Emotional Well-Being tool^b^**		
	Okay	18
	Good	2
	Not so good	5
**Visits to given emotion from the Emotional Well-Being tool, n^b^**		
	Grief	6
	Relief	6
	Supported	4
	Sadness	2
	Guilt	3
	Regret	1
	Shame	1

^a^IUD: intrauterine device.

^b^Returning and 1-time visitors.

**Table 2:** Number of page views for myPostCare.

myPostCare pages	Values, n	
Postprocedure Care	46	
Emotional Well-Being	28	
Contraception Explorer	27	
Postprocedure FAQs	25	
Emotional Support Tool	21	
Sexual Health	21	
Abortion Myth or Fact Quiz	15	
Resources	13	
Menstrual Cycle 101	12	
Meditation 101: Meditation for Beginners	10	
Book an Appointment	6	
Dealing with Difficult Feelings	6	
About Us	5	

### Email Notifications Analytics

**Figure 2:**
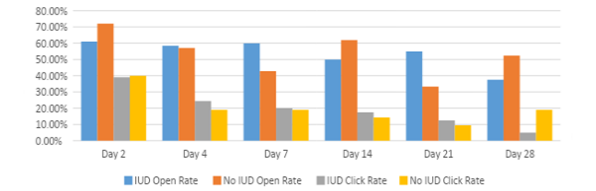
Comparison of email open and click rates for intrauterine device (IUD) versus no IUD stream in percentage.

Among the 62 participants enrolled, 2 (3%) unsubscribed from email notifications after the “Welcome Message” on day 0, and 2 (3%) participants’ email address was not valid. The average open rate was 80%, and the click rate was 36%. The highest open and click rates were for Welcome Message at 73.1% and 31.3%, respectively. Interestingly, the desktop device was 57.4% and mobile was 42.6%, which is different from the device from which the website was viewed. The email open rates were higher than the click rates throughout. The open rates declined for both IUD and no IUD over time; however, they remained stable at an average of 53.7% and 53.8%, respectively. Figure 2 graphically represents data of the *IUD* versus *no IUD* streams open and click rates for given days.

## Discussion

### Principal Findings

myPostCare is the first comprehensive web-based postabortion tool in Canada and has the potential to be integrated as part of family planning services. Integration of myPostCare into clinical practice provides an opportunity to consider a new approach to supplement follow-up care for abortion care specifically but women’s health generally. This study demonstrates the design and development of a comprehensive mobile intervention to facilitate care for women and people who can become pregnant and undergo a procedural abortion to support and normalize the emotional and physical aspects after abortion. We used a human-centered design methodology, an iterative development process that was informed by input from key stakeholders such as patients, family planning experts, and administrators involved in abortion care [22,37,38]. The results from the pilot evaluation of myPostCare demonstrated that it was feasible, acceptable, and satisfactory for women and people who can become pregnant.

Specifically, this 3-phase study demonstrates the importance of including the end users and key stakeholders in the design, development, and evaluation of a mobile intervention that services a population and health care issue that continue to be stigmatized. Formative research has provided important information regarding women’s interactions with technology, their needs and desires around follow-up and access to information, and feedback on design, which is essential for the success of myPostCare. A unique finding of this study that was supported in the literature was the importance of including a component of emotional support as part of follow-up abortion care [39]. Furthermore, we learned that the success of myPostCare was not only owing to the interactive tools and information provided by the website but that the appropriately timed automatic email notifications that women received was an important aspect of their care throughout the 30 days after the procedure. An iterative design process was important to ensure that the research team was continually evaluating that myPostCare realized the needs of the target users.

We adopted a few theoretical frameworks, all of which use a comprehensive participatory approach to developing eHealth technologies. This was similarly performed by Gilbert et al [38] in the development of Get Checked Online, a web-based sexually transmitted infection testing resource. More specifically, integrating the Technology Acceptance Model and Theory of Reasoned action with the human-centered design methodology, we used a holistic approach to developing myPostCare. According to the Technology Acceptance Model, perceived ease of use and perceived usefulness of a system are the 2 predominant indicators of system adoption [27,32]. Participants in our study were accustomed to using some form of technology, either mobile phones or computers; did not require acquisition of new skills; and were keen on developing a technology-based tool to support follow-up care after an abortion. Importantly, myPostCare will not eliminate structural barriers to comprehensive abortion care, and although it may not directly affect health behavior and decision-making, it may assist in making the delivery of abortion care more efficient, convenient, patient centered, and accessible.

myPostCare is a unique addition to the literature because of its methodology and outcome. There is evidence to support eHealth technologies to improve health care; however, currently there is limited research on mobile interventions specifically to address postabortion care, although there are various interventions for contraception use. A randomized trial in Cambodia demonstrated that involving women in the design and testing of a mobile intervention to support postabortion contraception led to more women in the intervention group reporting use of effective contraception at 4 months; specifically, the use of long-acting contraceptives was higher in the intervention group at 4 and 12 months after the procedure [40]. Previous feasibility trials focused on usability and acceptability have highlighted the importance of conducting a pilot study first, which can then assist with the design of a larger randomized trial to measure effectiveness [41]. Finally, similar to studies on the development and testing of contraception tools, the integration of evaluation in real-time clinical care is essential to ascertain the barriers and challenges to implementation in the future [22].

The limitations of this study include overall generalizability to other populations, small convenience sample sizes for all 3 phases, loss to follow-up and low response rates in this challenging population, and recruitment bias. The sample size of 6 participants in the qualitative interviews was small, ideally requiring 20 participants to achieve meaningful saturation. Given that this study is an extension of 2 previous phases, researchers felt confident in the analysis being generalizable compared with the findings of the 2 previous phases and from previous studies highlighting the type of gaps that myPostCare fills as per the participants’ reflections. As it pertains to recruitment bias, those who consented to participate were likely individuals who are more engaged with technology, have higher socioeconomic demographics, and are more likely to be early adopters of a digital health intervention to support abortion care. In previous studies, this is referred to as a Digital Divide, which suggests that although many developers of technology-based health interventions are optimistic about their impact; this needs to be balanced by the fact that the pattern of adoption is along social gradients [38]. New technologies such as myPostCare may further reinforce these social divides. Furthermore, abortion continues to be a stigmatized issue, which can be a limitation for research, as this can be a sensitive topic for most and posed difficulties with recruitment and loss to follow-up in our study. We evolved throughout each phase of the study to consider the challenges faced with patient engagement. For instance, recruitment took longer than expected for the qualitative interviews. We assumed that lack of participant engagement may be associated with stigma about abortion. In addition, we recognized that conducting research immediately after the procedure might be a sensitive time for individuals. This will need to be taken into consideration for future studies, particularly when thinking about diversifying the participants recruited and obtaining robust response rates for analysis.

Balancing these limitations are the strengths of our study, including the successful development of human-centered design elements, wide stakeholder engagement, diverse expertise on the research team, a large proportion of our sample size that was from rural locations, rigorous research methodologies, iterative design process, and development of the first web-based postabortion tool in Canada.

Further research could involve evaluating the effectiveness of myPostCare.ca and the overall patient experience through a randomized controlled trial. In addition, as suggested in other web-based literature [38], a health equity impact assessment with expert consultation and literature review may also help identify ways in which myPostCare reinforces or alleviates health inequities in sexual health services.

### Conclusions

myPostCare was found to be feasible and acceptable to women and people who can become pregnant to support follow-up care after a procedural abortion. There are obvious digital divides in health care specifically, as there are limited digital tools for women’s health in Canada. Thus, there is great potential for expansion of myPostCare. More specifically, since the introduction of Mifepristone in Canada, the first area of expansion will be for medication abortion. Generally, the expansion may then involve other aspects of women’s reproductive health.

We learned that key stakeholder engagement and understanding the organizational context are important. These factors are important for ongoing research initiatives and their implementation in clinical practice. Engaging stakeholders and potential users in a participatory process throughout the entire design and development of myPostCare was crucial to its success. Applying an iterative design and evaluation process that was flexible and dynamic, considering the factors of implementation at the outset, keeping in mind how myPostCare could change health care delivery, and the use of a multidisciplinary team were all unique and important aspects.

This study demonstrated that a technology-based intervention for postabortion care is feasible and acceptable. The success of myPostCare was based on the incorporation of a multidisciplinary team; participatory user-centered design process; robust stakeholder engagement; and the provision of nonjudgmental, nondirective, and medically accurate information. This study provides an example of the ongoing development of technology-based family planning services and is aligned with a larger gender-equitable, evidence-based programmatic agenda in Canada.

## Appendix

Multimedia Appendix 1 Demographic data.

## Abbreviations

IUD: intrauterine device
mHealth: mobile health
SUS: system usability scale

## Data Availability

Qualitative and quantitative data are available upon request; however, the data will be destroyed 10 years after its collection for this study.
