# Supplementary material for: Feasibility and Acceptability of a Mobile Technology Intervention to Support Postabortion Care After Surgical Abortion (the FACTS Study Phase 3): Mixed Methods Prospective Pilot Study
Source: JMIR Form Res. 2024 Jan 9;8:e46284. doi: 10.2196/46284 (PMC10807380; doi:10.2196/46284)
Supplement: Multimedia Appendix 1 [file formative_v8i1e46284_app1.pdf]

	Total		group		
			responded to follow up	did not respond to follow up	
	No. 62		No. 25	No. 37	P-value
**Age**					
Median (IQR)	30 (25 - 34)		29 (25 - 33)	30 (24 - 34)	0.99
**Born in Canada**					
Yes	37 (59.7%)		16 (64.0%)	21 (56.8%)	1
No	19 (30.6%)		8 (32.0%)	11 (29.7%)	
Missing	6 (9.7%)		1 (4.0%)	5 (13.5%)	
**How far travelled to the clinic**					
10 km or less	22 (35.5%)		8 (32.0%)	14 (37.8%)	0.78
10 to 50 km	25 (40.3%)		12 (48.0%)	13 (35.1%)	
50 to 100 km	4 (6.5%)		1 (4.0%)	3 (8.1%)	
>100 km	9 (14.5%)		3 (12.0%)	6 (16.2%)	
Missing	2 (3.2%)		1 (4.0%)	1 (2.7%)	
**Highest level of Education**					
Elementary school, middle school, some high school	4 (6.5%)		2 (8.0%)	2 (5.4%)	0.65
High school graduation	20 (32.3%)		7 (28.0%)	13 (35.1%)	
Trades certificate or Non-university certificate or University certificate below bachelor's level	17 (27.4%)		6 (24.0%)	11 (29.7%)	
Bachelor's degree	13 (21.0%)		6 (24.0%)	7 (18.9%)	

Post graduate degree or professional qualification (e.g. MSc, PhD)	6 (9.7%)		4 (16.0%)	2 (5.4%)	
Prefer not to answer	2 (3.2%)		0 (0.0%)	2 (5.4%)	
**Income**					
$0 - $14,999	12 (19.4%)		6 (24.0%)	6 (16.2%)	0.82
$15,000 - $24,999	8 (12.9%)		5 (20.0%)	3 (8.1%)	
$25,000 - $34,999	7 (11.3%)		2 (8.0%)	5 (13.5%)	
$35,000 - $44,999	8 (12.9%)		3 (12.0%)	5 (13.5%)	
$45,000 - $54,999	7 (11.3%)		2 (8.0%)	5 (13.5%)	
Above $55,000	11 (17.7%)		4 (16.0%)	7 (18.9%)	
Prefer not to answer	9 (14.5%)		3 (12.0%)	6 (16.2%)	
**Arizona Integrative Outcome scale**					
Mean (SD)	51.50 (±21.85)		45.14 (±17.79)	55.88 (±23.52)	0.068
Missing	8 (12.90%)		3 (12.00%)	5 (13.51%)	
**WHO-5 wellbeing index**					
Mean (SD)	44.6 (±25.1)		43.2 (±20.5)	45.6 (±27.9)	0.64
Missing	2 (3.2%)		1 (4.0%)	1 (2.7%)	

Table 1. Demographic Data
